# Health literacy – study protocol for LiSa cohort study

**DOI:** 10.1186/s12889-024-19148-8

**Published:** 2024-06-29

**Authors:** Maria João Batalha, Tiago Gabriel, Ana Valentim, Ana Soledade, Cátia Gomes, Bartolomeu Alves, Estêvão Soares dos Santos, Rui Passadouro, Sara Simões Dias

**Affiliations:** 1 ciTechCare – Center for Innovative Care and Health Technology, Polytechnic of Leiria, Leiria, Portugal; 2 Unidade Local de Saúde da Região de Leiria, Leiria, Portugal; 3 Câmara Municipal de Leiria, Leiria, Portugal; 4 School of Health Sciences, Polytechnic of Leiria, Leiria, Portugal; 5 Departamento Local de Saúde Pública do Oeste, Unidade Local Saúde Oeste, Caldas da Rainha, Portugal

**Keywords:** Health literacy, Anxiety and depression, Metabolic risk, Health related behaviors, Cohort study

## Abstract

**Background:**

Health literacy is the degree to which individuals have the ability to find, understand, and use information and services to inform health-related decisions and actions for themselves and others, whether at home, at the workplace, in the community, marketplace, healthcare sector, or the political arena. The main aim of this project is to measure health literacy in the adult population living in the municipality of Leiria over the next 10 years. As secondary objectives it is intended to characterize anxiety and depression, metabolic risk and health behaviors in the same population and over the same period.

**Methods:**

This is a prospective cohort study that collects data on HL, anxiety and depression, health characteristics, health behavior and sociodemographic data. The study population will be composed by adults (≥ 18 years old) who are non-institutionalized and living in private households in Leiria. The random sample is stratified by gender and age groups. A face-to-face interview will be conducted with the Computer Assisted Personal Interview at baseline. Follow-up will be carried out every 2 years via telephone call. The association between independent variables and health literacy is examined by means of variance analysis with measurement repetition, and taking into consideration follow-up.

**Discussion:**

The LiSa project is a population-based study, derived from a random sampling technique that will allow the analysis of health outcomes in a representative sample of the population of the municipality of Leiria. The LiSa study will be a valuable resource for epidemiological research, as it will provide fundamental information to improve public health policies regarding health literacy in Portugal.

**Trial registration:**

Clinical trials: NCT05558631 (registered on 26/09/2022).

**Supplementary Information:**

The online version contains supplementary material available at 10.1186/s12889-024-19148-8.

## Background

According to the World Health Organization (WHO), health is considered “a state of complete physical, mental and social well-being and not merely the absence of disease or infirmity” [1]. Currently, health constitutes an essential axis of development that crosses and involves all sectors of activity, implying the dissemination of health as policies and the active participation of all citizens.

Health literacy (HL) is a topic that has been gaining increasing importance over the last few decades [2]. HL involves the knowledge, motivation and skills to access, understand, evaluate and apply health information in ways which promote and maintain good health [3]. Citizens with a higher level of HL find it easier to make everyday decisions related to healthcare, disease prevention, and health promotion to maintain or improve quality of life [4]. In recent decades in Portugal, the promotion of citizens’ HL has been identified as the way to improve health care, emerging as a concern in the definition of health policies. This is because different studies have shown that an inadequate level of HL can have significant implications for health outcomes, the use of health services and, consequently, health spending [5].

Studies indicate that the incidence of mental illness is increasing, with anxiety and depression being among the most common illnesses in the community [6, 7]. Patients with depression typically experience symptoms of anxiety, and those with symptoms of anxiety also experience depression. It can be difficult to discriminate between these two conditions, however both depression and anxiety disorders require specific and appropriate treatment, as they are associated with significant morbidity and mortality [7].

The gap between individuals who need mental health care and those who receive appropriate care is also a concern, as up to 70% of individuals who need mental health treatment fail to access the services they need [8]. Barriers to access treatments include lack of access to services, the potential client’s limited overall understanding of therapy treatment, and the perception that therapy treatment is too demanding or not relevant to the presenting concerns. These barriers are directly related to (lack of) HL [9].

Given these issues, it is necessary educate about the disease, focusing on the individual drivers which enables empowering citizens with knowledge to make conscious and informed decisions about the promotion of individual and collective health.

In 2021, the Municipality of Leiria prepared the Municipal Health Strategy, a document that serves as a tool to guide the development of health policies and intervention strategies of the Municipality. The document’s SWOT analysis presented HL and mental health as one of the weaknesses in the municipality, while hypertension and diabetes were identified as the leading health problems [10]. The Municipal Health Strategy is a strategic document, aligned with the main documents in the health area, namely the National Health Plan [11] where one of the main objectives are the reduction of alcohol consumption and exposure to tobacco.

Thus arises the LiSa study, a cohort study in Leiria, where citizens will be the essential source of information. The main aim of this project is to measure health literacy in the adult population living in the municipality of Leiria over the next 10 years. Concurrently, it is intended to characterize anxiety and depression and metabolic risk in the same population and over the same period.

## Methods

### Study design

The study is being developed and implemented by a multidisciplinary, multicenter team (researchers, health professionals, designers, mayors, among others). It is a prospective, longitudinal, closed cohort study over the next ten years with five data collection points. The guidelines from observational studies in epidemiology (STROBE) were applied to the study design [12]. The first data collection (baseline) will be carried out through an individual door-to-door interview, with a pre-established route, while the remaining data collection points will be managed via telephone.

To account the expected loss of participants to follow-up over time, we interviewed experts in cohort studies and potential study participants with the intention of helping and advising us in developing the study design. For that we used service design methodologies, namely informal interviews, and user groups definition.

### Study population

The study population will be composed by adults (≥ 18 years old) who are non-institutionalized and living in private households in Leiria. Exclusion criteria will be: residents in hospitals, nursing homes, military barracks, or prisons, and residents unable to speak Portuguese or with a complete inability to respond to the questionnaire, either directly or through a person living with them [13].

### Sample size

Residents of the municipality of Leiria aged 18 years or more. A sample size of 4003 (2001 males and 2002 females) were determined. A random sample stratified by gender and age groups (18–29, 30–64, 65 or more), for a confidence level of 95% and margin of 5% error was computed, in this case the sample need to collect should be 2287 participants. However, from previous studies [14] it is known that there is a high dropout rate in longitudinal studies, reaching approximately 50% in the 3rd wave. As if pretending to make 5 waves, the sample was increased by 75%. In other words, considering a dropout rate of 75%, data from 4003 participants should be collected. See Table 1.

**Table 1:** Sample size of LiSa cohort study

	Leiria’s population		Sample size		+ 75% dropout	
	Male	Female	Male	Female	Male	Female
18–29 years	9367	9165	380	380	665	665
30–64 years	30,295	32,625	383	383	670	670
65 ou + years	9632	12,404	380	381	665	667
**Total**	**49,294**	**54,194**	**1143**	**1144**	**2001**	**2002**

### Recruitment phase: face-to-face interview

The team of interviewers that will carry out the baseline data collection will be composed of 32 interviewers. Prior to the start of the study, all participating interviewers will be trained, by Lisa team, to standardize procedures. This training will cover the study protocol, and topics related to coaching, study design, study characteristics, logistics, ethical and legal issues, sample selection methodology, interview procedures (exercises of roll-play) and software. Only the candidates that successfully do this training will be selected to the interviews face-to-face, to assure the quality of the data collection [15].

Interviewers must be motivated and know very well the study and its objectives. For that, an interviewer’s manual/guide was designed with all the details of the study. The interviewers are grouped in teams of four and the interviews will be done by two interviewers, a male and a female. In each team of four, there is an interviewer coordinator who will manage the interviewer teams (scheduling interviews, travel, etc.), manage conflicts that may arise and make sure that the interviewers’ interaction with the participants is carried out correctly and according to the script.

### Data collection

At baseline a face-to-face interview will be conducted with the Computer Assisted Personal Interview (CAPI) system: all interviewers have a tablet with the software which will provide the questionnaire. The questionnaire was designed by LiSa’s research team. Both the survey and software performance were tested in a small sample of subjects, and results validated by the LiSa research team before being used by the interviewers. It is estimated it will take approximately 30 min to complete the survey.

Since there is no reliable list of households in Portugal, a random selection of points in the map of each location will be performed. Addresses will be selected, and afterwards the “random walks” will start. Interviewers will register each selected address and, if someone is at home, they will collect information on age and gender of the different people living in that address, and will give information about the study. After validation of the selected address and inhabitants, the interviews will be scheduled, or if the person is available will be immediately asked to answer the questionnaire. In order to assure the successful data collection, up to three visits to each address (one during the weekend) will be made [13]. At each address, the person whose birthday is closest to the day of the visit (aged 18 years or older) will be selected for the interview.

Between February and April 2023, five interviewers conducted a pilot study, in the largest parish in the council, with the aim of testing the proposed methodology. Taking into account that data collection went well, we will maintain this data collection methodology and start the first data collection in February 2024.

### Follow-up

The collection of new data will be carried out every 2 years via telephone call.

One of the approaches to mitigate the abandonment of participants (loss to follow-up) is to ensure good communication between the study team (interviewers, researchers, health professionals, designers, mayors) and cohort participants, using effective communication channels, updated, and provide quick feedback on health information to the participant.

The reasons why participants drop out of the study will be documented in order to analyze the bias of the study results.

### Communication of the study

Communication about the LiSa study is very important, the population will more easily join the study if they know the objectives and long-term benefits of the study. Thus, population will learn about the LiSa study in several ways. Outdoors and advertising boards will be spread across the municipality. The screens at the city’s main hospital and health centers will have information about LiSa study. There will be LiSa’s flyers in the pharmacies, health units, coffees, bookstores, parish council and others public places. One week before the interviewers knock on people’s door, flyers will be distributed in the mailbox with dates and times when interviewers will walk in that area. The priests will also announce at Mass when the interviews will take place in that parish.

### Variables under study

At baseline, HL, anxiety and depression, health characteristics (metabolic risk, auto-report chronic diseases, anthropometric variables), lifestyle habits (tobacco, alcohol, physical activity) and sociodemographic characteristics are collected from each participant.

### Health literacy

HL will be measured through European Health Literacy Survey Questionnaire short-form version (HLS_19_-Q12). To measure HL in an adequate way for public health-oriented surveys of general population in Europe, the Health Literacy Survey (HLS-EU) was conducted in 2012 [16] using a new measurement tool developed from the conceptual model of Sørensen et al. [17]: the “47-item European Health Literacy Survey Questionnaire” (HLS-EU-Q47) [18]. HLS-EU was developed to measure HL in populations [18] based on a conceptual framework reflecting four information-processing dimensions (i.e., accessing, understanding, appraising, and applying) within three health domains (i.e., health care, disease prevention, and health promotion) [17]. Each item in the questionnaire assesses the perceived difficulty of a specific health-related task for the respondent. Since 47 items were excessively time-consuming for some HL researchers’ short forms have been validated in several languages to be used in the European Health Literacy Survey [4, 19, 20] The HLS_19_-Q12 was translated and validated in a previous study in Portuguese population [3]. The scale contains 12 items that measure HL. Each item assesses the perceived difficulty of completing a specific health-related task by asking “On a scale from very difficult to very easy, how easy would you say it is to [e.g, find information on symptoms of illnesses that concern you]?” Each item is rated on a 4-point Likert-type scale (1, very difficult; 2, fairly difficult; 3, fairly easy; 4, very easy). To correctly assess respondents’ health literacy, participants must answer at least 10 of the 12 questions. The general HL index (G-HL12) is calculated as follows: G-HL12 index = (mean − 1) x (50/3). Four levels of HL are defined: inadequate (0–25), problematic (25.1–33), adequate (33.1–42), and excellent (42.1–50) [21].

### Anxiety and depression

To evaluate symptoms of anxiety and depression we applied the Hospital Anxiety and Depression Scale (HADS) Portuguese validated version [22]. The HADS was originally developed by Zigmond and Snaith [23] as a screening tool to apprehend clinically significant states of anxiety and depression in a non-psychiatric hospital setting. Individual anxiety and depression scores were calculated by summation of the appropriate seven items and thus can range from 0 to 21, with higher scores indicating higher levels of anxiety or depression, respectively. In both subscales, a score between 0 and 7 is “normal,” between 8 and 10 “mild,” between 11 and 14 “moderate,” and between 15 and 21 “severe” (idem). Presence of anxiety and depression symptoms was defined when HADS scale was ≥ 11, since Snaith suggested a score ≥ 11 was indicative of “caseness” to a mood disorder [24]. This scale is used in a lot of observational studies [14, 25].

### Health characteristics

Metabolic risk is assessed by the Finnish Diabetes Risk Score-FINDRISC (FINDRISC). FINDRISC is a simple and practical tool originally designed in Finland to identify individuals at high risk of type 2 diabetes mellitus onset, without the need for laboratory tests [26, 27]. The score is based on eight easily identifiable parameters (age, body mass index, waist circumference, hypertension, physical activity level, diet, occurrence of previous hyperglycemia, and family history of diabetes) and provides a measure of the probability of developing diabetes over the next 10 years. This score is being used in different European populations [28, 29].

Participants will be asked if they had been previously diagnosed with some chronic disease (high cholesterol level, high blood pressure, heart disease, diabetes, and pulmonary disease). Self-reported abdominal circumference, height and weight and based on these data, body mass index (BMI, weight/height2, in kg/m2) will be calculated and categorized according to the World Health Organization classification in four categories: underweight (BMI < 18.5 kg/m2), normal (BMI between 18.5 and 24.9 kg/ m2), overweight (BMI between 25 and 29.9 kg/m2), and obesity (BMI ≥ 30 kg/m2) [30].

### Health related behavior

Health related behavior covers the following domains: alcohol intake (daily, occasionally, and never), consumption of fruits and vegetables, smoking habits, and physical activity.

Alcohol intake is measure by Alcohol Use Disorders Identification Test (AUDIT) developed by WHO, it is a simple and effective method of screening for unhealthy alcohol use, defined as risky or hazardous consumption or any alcohol use disorder [31].

Smoking habits (current smoker, past smoker, and never smoked) is accessed by Fagerström Test for Nicotine Dependence. This is a standard instrument for assessing the intensity of physical addiction to nicotine. The test was designed to provide an ordinal measure of nicotine dependence related to cigarette smoking. It contains six items that evaluate the quantity of cigarette consumption, the compulsion to use, and dependence [32, 33]. 

### Study validity

Data collection forms will be further monitored centrally to check for missing data or inconsistencies, including potential participant telephone contact to confirm the answers. Also, a monthly analysis of the collected data will be carried out to monitor its quality. This statistical analysis will be carried out at different levels: by teams of interviewers, by locations, age groups and gender, to minimize errors in the data.

The data collected will be stored on a specific server with a high level of security.

### Statistical analysis

Descriptive statistics will be computed to describe the baseline characteristics of the participants (gender, age, education and marital status). Normally distributed variables will be described by the mean and standard deviation. In the case of non-normal distributions, the data will be presented as median and interquartile range. For qualitative variables, absolute and relative frequencies will be calculated. At baseline comparisons between groups (e.g. with and without anxiety) will be undertaken using *t-*tests for continuous normally distributed variables or Wilcoxon for non-normally distributed variables. Chi-squared tests will be used for categorical variables, and Fisher’s Exact test will be used for categorical variables within smaller sample sizes.

Taking into consideration follow-up, the changes in HL will be evaluated by means of variance analysis with measurement repetition. After checking the statistical model pre-requisites, sociodemographic characteristics will be added by means of linear regression analysis in the final model.

For all calculations the level of statistical significance will be set to *p* < 0.05 and Stata software will be used.

## Discussion

The HLS-EU revealed that 47.6% of respondents in Europe had limited HL (insufficient or problematic), which is reflected in almost half of the people surveyed [16]. The HLS-EU-PT presents itself as an adequate instrument to assess the level of HL of the Portuguese population, it was applied throughout the national territory, including the autonomous regions, through researchers from an academic network. The results revealed that 61% of the Portuguese population surveyed had a problematic or inadequate level of general HL [5]. This problem must be overcome, because at some point in our lives, we all need to be able to find, understand and use health information and services [34]. Low levels of HL are related to a lower ability to manage chronic diseases, greater number of hospitalizations, incorrect use of emergency services and infrequent use of preventive practices [35]. Considering the impact of HL levels on individual and collective health, on the use of health services and, consequently, on health spending, the development of diagnostic tools for the HL level of populations has become crucial [5, 36–38].

The LiSa study is a cohort study being implemented in Leiria by a multidisciplinary, multicenter team (epidemiologists, doctors, nurses, designers, mayors, among others) and aims to characterize HL in the adult population of the municipality of Leiria over the next 10 years, with measurements every 2 years. It is also intended to characterize the levels of anxiety and depression, metabolic risk, alcohol and smoking in the adult population, since these are problems very present in today’s society. Most of the existing studies that look for HL in Portugal are based on cross-sectional examinations [3, 5]. Longitudinal studies have advantages in terms of the quantity or quality of the data that collected, details of the life course, establishing the order in which events occurs and reduce recall bias.

The results of this study will be published in well-known international journals, conferences and congresses. Project’s data will be used in national and international dissertations on condition on referral to the cohort. The research results will be present to national decision-makers in oral and written form. The LiSa study has already been presented and announced to Leiria’s community and in the social media.

### Electronic supplementary material

Below is the link to the electronic supplementary material.

Supplementary Material 1

## Data Availability

The datasets used and/or analysed during LiSa study will be available from the corresponding author on reasonable request.
