# Supplementary material for: Health literacy – study protocol for LiSa cohort study
Source: BMC Public Health. 2024 Jun 29;24:1737. doi: 10.1186/s12889-024-19148-8 (PMC11218293; doi:10.1186/s12889-024-19148-8)
Supplement: Supplementary file 1 — Supplementary Material 1 [file 12889_2024_19148_MOESM1_ESM.docx]

**HLS_19_-Q12**

**On a scale from very easy to very difficult, how easy would you say it is to…**

1. Find information on treatments of illnesses that concern you?
2. Understand what to do in medical emergencies?
3. Judge the advantages and disadvantages of different treatment options?
4. Follow the instructions on medication?
5. Find information on how to manage mental health problems like stress and depression?
6. Understand why you need health screenings?
7. Judge if the information on health risks in the media is reliable?
8. Decide how you can protect yourself from illnesses based on advice from family and friends?
9. Find information on health attitudes such as exercise, healthy food and nutrition?
10. Understand information on food packaging?
11. Judge which everyday behaviors are related to health?
12. Make decisions to improve your health?

**Numa escala de muito fácil até muito difícil, quão difícil é para si..**

1. Descobrir onde obter ajuda especializada quando está doente?
2. Compreender o que fazer numa urgência médica?
3. Avaliar as vantagens e desvantagens de diferentes opções de tratamento?
4. Seguir as instruções do seu médico ou farmacêutico?
5. Encontrar informação para lidar com problemas de saúde mental?
6. Compreender informação sobre rastreios ou exames recomendados?
7. Avaliar quão segura é a informação sobre hábitos pouco saudáveis, como fumar, atividade física insuficiente ou tomar bebidas alcoólicas em demasia?
8. Decidir como pode proteger-se da doença com base em informação dada através dos meios de comunicação?
9. Encontrar informação sobre estilos de vida saudáveis, como a prática de exercício físico, alimentação saudável ou nutrição?
10. Compreender conselhos sobre saúde que lhe chegam da sua família e amigos?
11. Avaliar de que modo as condições da sua habitação podem afetar a sua saúde e bem-estar?
12. Tomar decisões para melhorar a sua saúde e bem-estar?
